# Longitudinal Associations of Mental Disorders With Physical Diseases and Mortality Among 2.3 Million New Zealand Citizens

**DOI:** 10.1001/jamanetworkopen.2020.33448

**Published:** 2021-01-13

**Authors:** Leah S. Richmond-Rakerd, Stephanie D’Souza, Barry J. Milne, Avshalom Caspi, Terrie E. Moffitt

**Affiliations:** 1 Department of Psychology, University of Michigan, Ann Arbor; 2 Centre of Methods and Policy Application in the Social Sciences, University of Auckland, Auckland, New Zealand; 3 Department of Psychology and Neuroscience, Duke University, Durham, North Carolina; 4 Center for Genomic and Computational Biology, Duke University, Durham, North Carolina; 5 Institute of Psychiatry, Psychology, and Neuroscience, King’s College London, London, England; 6 Promenta Center, University of Oslo, Oslo, Norway

## Abstract

**Importance:**

Excess risk of physical disease and mortality has been observed among individuals with psychiatric conditions, suggesting that ameliorating mental disorders might also be associated with ameliorating the later onset of physical disability and early mortality. However, the temporal association between mental disorders and physical diseases remains unclear, as many studies have relied on retrospective recall, used cross-sectional designs or prospective designs with limited follow-up periods, or given inadequate consideration to preexisting physical illnesses.

**Objective:**

To examine whether mental disorders are associated with subsequent physical diseases and mortality across 3 decades of observation.

**Design, Setting, and Participants:**

This population-based cohort study used data from the New Zealand Integrated Data Infrastructure, a collection of nationwide administrative data sources linked at the individual level, to identify mental disorders, physical diseases, and deaths recorded between July 1, 1988, and June 30, 2018, in the population of New Zealand. All individuals born in New Zealand between January 1, 1928, and December 31, 1978, who resided in the country at any time during the 30-year observation period were included in the analysis. Data were analyzed from July 2019 to November 2020.

**Exposures:**

Nationwide administrative records of mental disorder diagnoses made in public hospitals.

**Main Outcomes and Measures:**

Chronic physical disease diagnoses made in public hospitals, deaths, and health care use.

**Results:**

The study population comprised 2 349 897 individuals (1 191 981 men [50.7%]; age range at baseline, 10-60 years). Individuals with a mental disorder developed subsequent physical diseases at younger ages (hazard ratio [HR], 2.33; 95% CI, 2.30-2.36) and died at younger ages (HR, 3.80; 95% CI, 3.72-3.89) than those without a mental disorder. These associations remained across sex and age and after accounting for preexisting physical diseases. Associations were observed across different types of mental disorders and self-harm behavior (relative risks, 1.78-2.43; *P* < .001 for all comparisons). Mental disorders were associated with the onset of physical diseases and the accumulation of physical disease diagnoses (incidence rate ratio [IRR], 2.00; 95% CI, 1.98-2.03), a higher number of hospitalizations (IRR, 2.43; 95% CI, 2.39-2.48), longer hospital stays for treatment (IRR, 2.70; 95% CI, 2.62-2.79), and higher associated health care costs (*b* = 0.115; 95% CI, 0.112-0.118).

**Conclusions and Relevance:**

In this study, mental disorders were likely to begin and peak in young adulthood, and they antedated physical diseases and early mortality in the population. These findings suggest that ameliorating mental disorders may have implications for improving the length and quality of life and for reducing the health care costs associated with physical diseases.

## Key Points

**Question:**

Are mental disorders associated with subsequent physical diseases and early mortality?

**Findings:**

In this population-based cohort study of more than 2 million New Zealand citizens who were followed up across 3 decades, mental disorders were associated with the subsequent onset of physical disease, the accumulation of physical disease diagnoses and associated health care use and costs, and early mortality. These associations were observed across different psychiatric conditions, across men and women, and across the life span, and they remained after accounting for preexisting physical diseases.

**Meaning:**

This study’s findings suggest that ameliorating mental disorders in early life may have implications for improving the health and life span of a population and reducing the health care costs associated with physical diseases.

## Teaser

This cohort study uses data from the New Zealand Integrated Data Infrastructure database to examine the association of mental disorders with subsequent physical diseases and mortality among the New Zealand population over a 30-year observation period.

## Introduction

Two global trends are currently occurring that challenge the well-being of young people. First, the demographic characteristics of populations are changing; humans are living longer and having fewer children, leaving fewer young workers to support larger numbers of retirees.^1^ Second, workforce expectations are shifting. Young people entering the modern labor force are expected to have higher levels of education and more specialized skills than those in previous generations.^2^ These changes may be associated with higher levels of stress among young people, increasing the importance of having good mental health in early life.

Problems with mental health in early life might also be associated with problems with physical health in later life. Data suggest that the same people who experience psychiatric conditions when they are young experience age-associated physical diseases when they are older.^3,4^ Excess risk of physical disease and mortality among individuals with mental disorders has been detected in data from community surveys and outpatient and inpatient hospital records^5,6,7,8,9,10,11,12^ and in data across different countries^4,13,14,15,16^ and socioeconomic strata.^17,18^ These findings suggest that preventing mental disorders in youth might be associated with the prevention of physical diseases and disabilities in older adulthood. The timing of the development of mental and physical illnesses is consistent with this possibility, as mental disorders peak in young adulthood, while noninfectious physical diseases and neurodegenerative conditions peak later in life.^19^

Previous research provides important information about the associations between mental and physical health. However, most studies have used end-of-life measures of poor physical health, such as all-cause mortality, cause of death, or years of life lost.^7,10,13,14,15,16,20^ Estimates of earlier outcomes (such as onset of first physical disease or number of disease episodes) would inform prevention efforts. Furthermore, studies estimating associations between mental disorders and physical diseases have largely relied on cross-sectional designs or follow-up periods of less than 15 years, with longer follow-up reserved for studies of mortality outcomes.^15,21^ Some studies have relied on retrospective self-reports, which are susceptible to recall bias.^4^ These issues limit the ability to capture mental and physical health problems at their onset and evaluate their associations over the life span. In addition, few studies have attempted to describe the temporal order in which mental and physical health problems occur, leaving open the possibility of reverse causation (ie, that physical disease contributes to mental disorders rather than vice versa). In this study, we addressed these gaps by using population-wide administrative data from 2.3 million New Zealand citizens aged 10 to 60 years at baseline to identify associations between mental disorders and subsequent physical diseases and mortality across 3 decades. We identified all diagnoses of mental disorders and chronic physical diseases that were included in public hospital records and all deaths that were recorded during the 30-year period.

We tested 4 hypotheses. First, we tested the hypothesis that mental disorders antedate early death. If observed, this finding would suggest that ameliorating mental disorders might have implications for improving life span (ie, how long we live). Second, we tested the hypothesis that mental disorders antedate the onset of physical disease. If observed, this finding would suggest that ameliorating mental disorders might also be associated with improvements in health span (ie, how well we live). Third, we tested the hypothesis that associations between mental disorders and physical diseases are found across different psychiatric conditions. If observed, this finding would suggest that ameliorating any mental disorder in early life might be beneficial for health in later life. Fourth, we tested the hypothesis that associations with mental disorders extend to other measures of physical health problems, including the number of different physical conditions and hospital admissions that individuals accumulate, the length of stay in hospitals, and the associated health care costs. If observed, these findings would suggest that ameliorating mental disorders could have implications for reducing health care use and the costs associated with physical diseases. To address the possibility of reverse causation, we tested whether associations between mental disorders and physical disease outcomes remained after controlling for preexisting physical diseases.

## Methods

Data were from the New Zealand Integrated Data Infrastructure, a collection of deidentified whole-of-population administrative data sources linked at the individual level.^22,23^ Ethical approval was obtained from the University of Auckland Human Participants Ethics Committee. Output data underwent confidentiality review by Statistics New Zealand Tatauranga Aotearoa. Informed consent was not obtained per rule 11(2)(c)(iii) of the New Zealand Health Information Privacy Code,^24^ which, under certain circumstances, allows for anonymized health data to be used for research purposes without the authorization of the individual concerned. The New Zealand Ministry of Health has confirmed that these circumstances were met for use of the data included in the current study. This study followed the Strengthening the Reporting of Observational Studies in Epidemiology (STROBE) reporting guideline.

### Study Population

The study population included the 2 349 897 individuals aged 10 to 90 years who were born in New Zealand between January 1, 1928, and December 31, 1978, and who resided in the country for any period between the July 1, 1988, and June 30, 2018, fiscal years. We selected this age range to capture the periods of peak prevalence for both mental disorders and physical diseases. We divided the population into age groups based on decade of birth (1928-1937, 1938-1947, 1948-1957, 1958-1967, and 1968-1978).

We collected information about admissions to public hospitals over a 30-year period from records maintained by the New Zealand Ministry of Health. We determined primary diagnoses, external cause codes, and procedure codes for admissions based on the *International Statistical Classification of Diseases and Related Health Problems, Tenth Revision*, and the corresponding *International Classification of Diseases, Ninth Revision* diagnostic codes. We obtained data for 9 broad categories of mental disorders: substance use, psychotic, mood, neurotic, physiological disturbance, personality, developmental, behavioral, and unspecified. We also obtained data about self-harm behavior. We collected information about 8 physical diseases classified as chronic by the New Zealand Ministry of Health; these diseases comprised coronary heart disease, gout, chronic obstructive pulmonary disease, diabetes, cancer, traumatic brain injury, stroke, and myocardial infarction. Details of specific diagnoses and their coding are provided in eMethods 1 in the Supplement. We collected information about mortality from records maintained by the New Zealand Department of Internal Affairs.

We measured additional factors associated with physical health problems and subsequent health care involvement on the individual level, including (1) the number of different health conditions, (2) the number of hospital admissions, (3) the total length of stay (in days) in the hospital, and (4) the total health care cost (eMethods 2 in the Supplement). The health care cost measure was skewed and was therefore log transformed for analyses.

### Statistical Analysis

We used Poisson regression models with relative risks (RRs) and Cox proportional hazards models (with censoring for outmigration) to estimate the associations of mental disorders and physical diseases with mortality across the 30-year observation period. We estimated the associations with mortality among individuals who were (1) diagnosed with only a mental disorder, (2) diagnosed with only a physical disease, and (3) diagnosed with both a mental disorder and a physical disease and compared them with (4) individuals who were not diagnosed with any mental disorder or physical disease during the observation period. Because these groups were likely to differ in the number of hospitalizations they experienced, we controlled for the total number of hospitalizations during the observation period.

**Figure 1.**
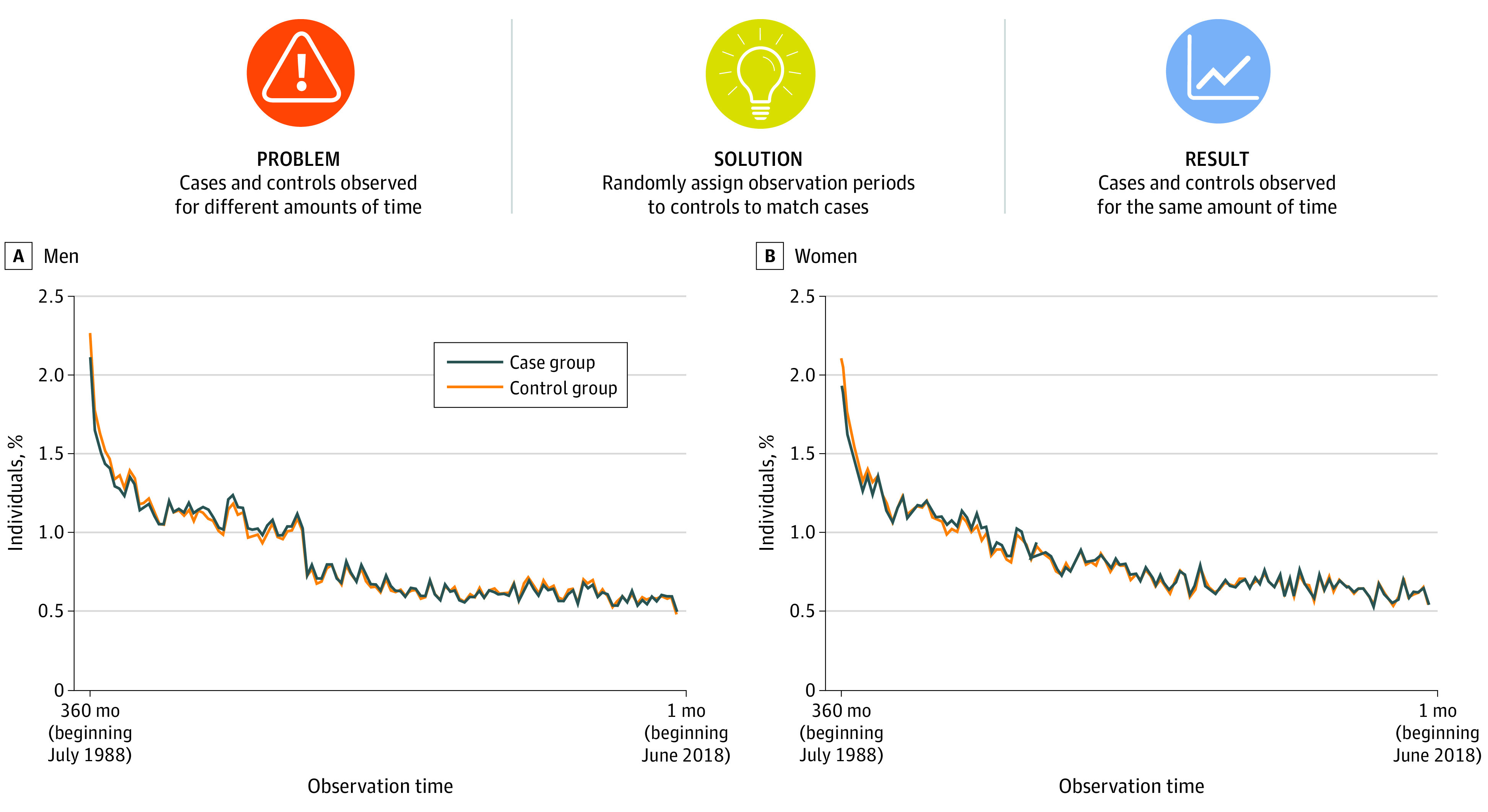
Random Matching Procedure To account for the different durations of observation time between individuals with a mental disorder (cases, observed from their first mental health hospitalization) and individuals without a mental disorder (controls, all observed from the start of the study period), we randomly assigned observation periods to controls to match observation durations among cases using hospital admission dates (month and year). Hospital admission dates were ascertained at the daily level, but cases and controls were matched based on the distributions of admission dates within each month. Matching was conducted within each birth cohort separately to account for cohort differences in the prevalence of mental health hospitalizations. Panels A and B show that the distributions of observation time among cases and their randomly-matched controls were similar. The percentages sum to 100% within cases and controls (within sex). In addition to random matching, we weighted the data based on time spent alive and in the country to account for any remaining differences between individuals in observation time owing to death or outmigration.

We used Poisson regression models with RRs and competing risks survival analysis (based on the risk of a physical disease vs death) to estimate the associations between mental disorders and subsequent physical diseases, controlling for physical diseases diagnosed before the individual’s first diagnosed mental disorder (the index disorder). To account for differing durations of observation between those with a mental disorder (observed from their first hospitalization for a mental disorder) and those without a mental disorder (observed across 30 years for all individuals without a mental disorder), we randomly assigned observation periods to the control group to match the observation durations among the case group (Figure 1; eMethods 3 in the Supplement). We weighted the data based on the duration of time that individuals were alive and residing in the country to account for any remaining differences in observation time between individuals owing to death or outmigration.

We used regression analysis to estimate the association between mental disorders and subsequent physical diseases and health care involvement, controlling for physical diseases diagnosed before the index mental disorder. We analyzed count outcomes (number of physical health conditions, number of hospital admissions, and number of days in the hospital [length of stay]) using negative binomial regression models with incidence rate ratios (IRRs). We analyzed continuously distributed outcomes (health care cost) using ordinary least-squares regression. Individuals in the case and control groups were matched on observation time, and data were weighted.

Associations were estimated by total population, birth period (age group), and sex. Models using the total population controlled for sex and birth year. Statistical significance was designated a priori as 2-sided *P* < .05. Because small associations can be statistically significant in large samples, we reported effect sizes and 95% CIs for all associations.

Per the confidentiality rules of Statistics New Zealand, reported counts were randomly rounded to a base of 3. Therefore, counts do not always sum to totals. Our analysis plan was preregistered and is available on the Statistics New Zealand website.^25^ Statistical analysis was performed using SAS Enterprise Guide, version 7.1 (SAS Institute), and all data were analyzed from July 2019 to November 2020.

## Results

**Figure 2.**
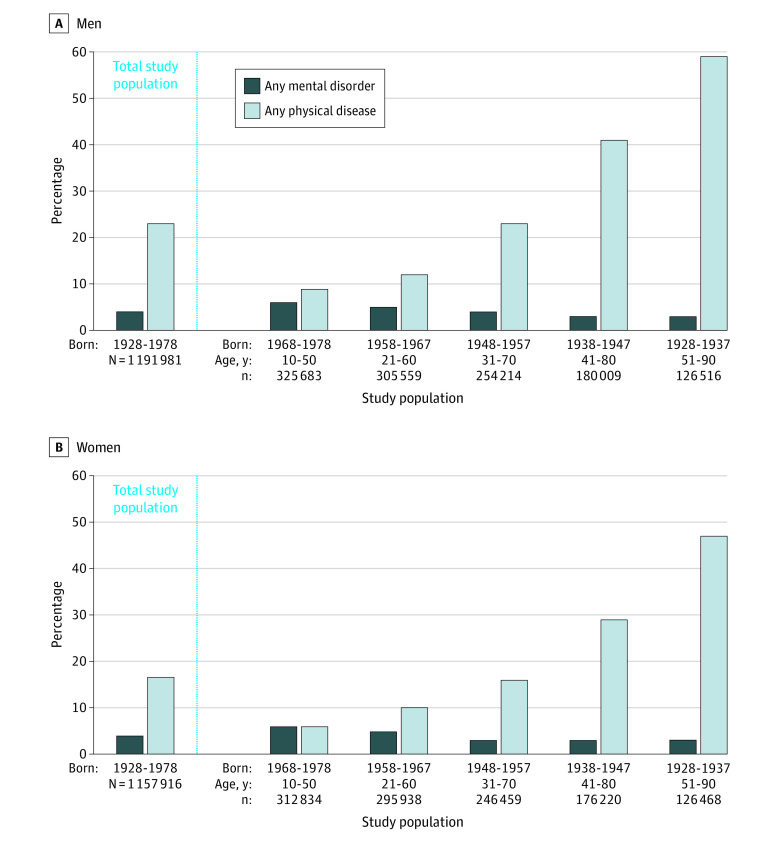
Distribution of Mental Disorders and Physical Diseases in the Study Population The figure shows the prevalence (based on inpatient hospitalization diagnoses) of individuals diagnosed with any mental disorder or any physical disease during the 30-year observation period. Counts were randomly rounded to a base of 3 per the confidentiality rules of Statistics New Zealand. Age ranges indicate ages during the 30-year observation period.

The study population included the 2 349 897 individuals (1 191 981 men [50.7%] and 1 157 916 women [49.3%]; age range at baseline, 10-60 years) who were born in New Zealand between 1928 and 1978 and who resided in the country for any period between July 1988 and June 2018. Of those, 126 516 men and 126 468 women were born between 1928 and 1937 (age range, 51-90 years), 180 009 men and 176 220 women were born between 1938 and 1947 (age range, 41-80 years), 254 214 men and 246 459 women were born between 1948 and 1957 (age range, 31-70 years), 305 559 men and 295 938 women were born between 1958 and 1967 (age range, 21-60 years), and 325 683 men and 312 834 women were born between 1968 and 1978 (age range, 10-50 years) (Figure 2).

During the 30-year observation period, 470 169 individuals (20.0%) were admitted to public hospitals for a physical disease, and 103 929 individuals (4.4%) were admitted to public hospitals for a mental disorder. Across the observation period, those diagnosed with a physical disease were more likely to be male than female (eg, 58.5% of men vs 47.1% of women born in 1928-1937; 9.1% of men vs 6.1% of women born in 1968-1978) and more likely to be older than younger (eg, 52.8% of individuals born in 1928-1937 vs 7.6% of individuals born in 1968-1978) (Figure 2). Similar or identical percentages of men vs women (eg, 3.0% of men and 3.3% of women born in 1928-1937; 6.1% of both men and women born in 1968-1978) and a higher percentage of younger vs older individuals (eg, 6.1% of individuals born in 1968-1978; 3.2% of individuals born in 1928-1937) were diagnosed with a mental disorder (Figure 2).

### Mental Disorders and Early Mortality

**Figure 3.**
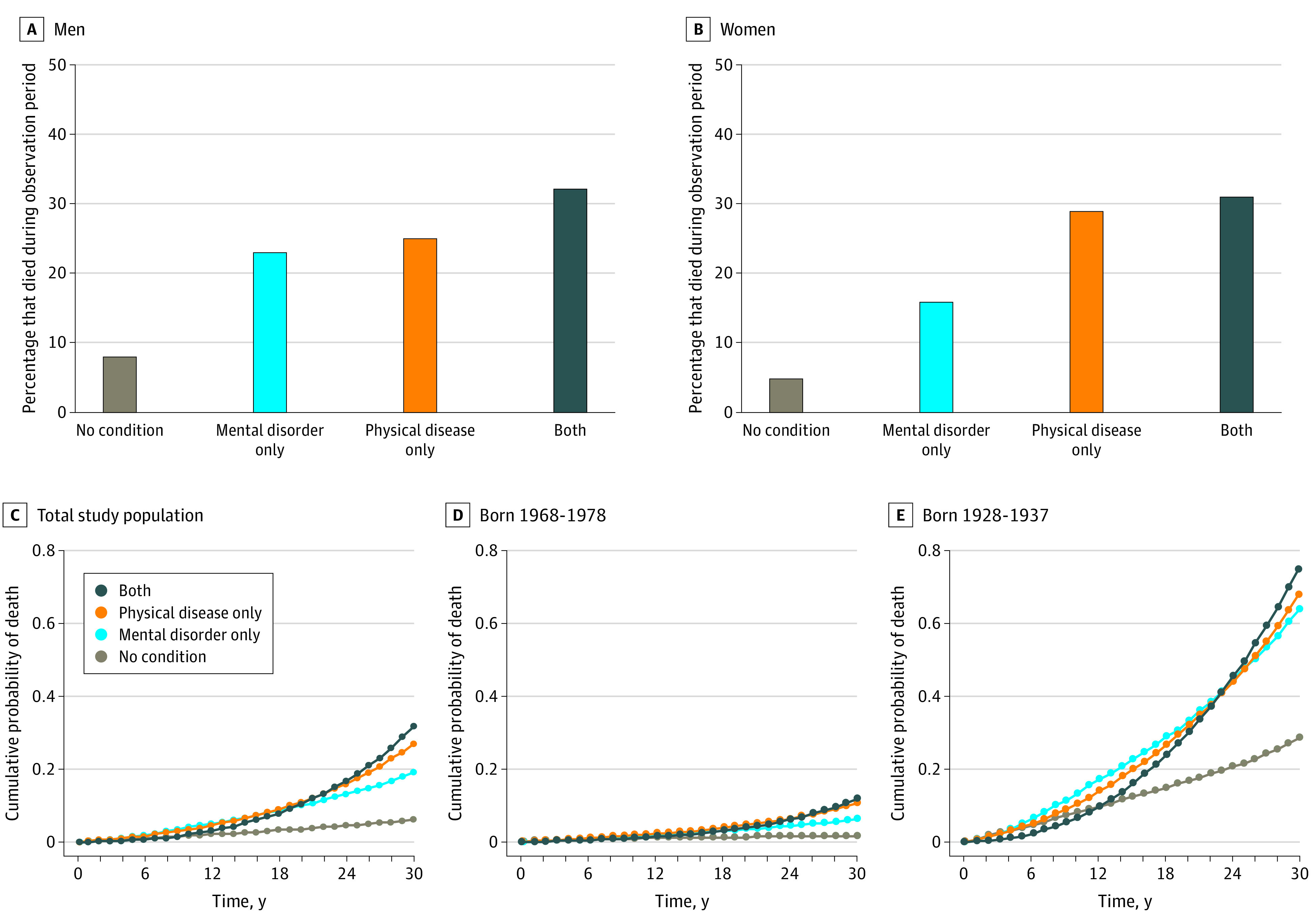
Associations Between Mental Disorders, Physical Diseases, and Mortality A, Estimates were age-standardized. A total of 885 558 men had no mental or physical condition, 35 454 men had mental disorders only, 253 155 men had physical diseases only, and 17 829 men had both mental disorders and physical diseases. B, Estimates were age-standardized. A total of 926 553 women had no mental or physical condition, 36 249 women had mental disorders only, 180 699 women had physical diseases only, and 14 409 women had both mental disorders and physical diseases. C, Estimates were age- and sex-standardized. D, Youngest cohort. Estimates were sex-standardized. E, Oldest cohort. Estimates were sex-standardized. Counts were randomly rounded to a base of 3 per the confidentiality rules of Statistics New Zealand. Hazard ratios for the associations between mental disorders, physical diseases, and mortality for varying intervals across the 30-year observation period are available in eTable 3 in the Supplement.

During the observation period, individuals with a physical disease were more likely to die (RR, 4.12; 95% CI, 4.08-4.15; *P* < .001) and more likely to die at younger ages (hazard ratio [HR], 4.69; 95% CI, 4.65-4.73; *P* < .001) compared with individuals without a physical disease or mental disorder (Figure 3; eTable 1 in the Supplement). An increased risk of mortality (RR, 3.39; 95% CI, 3.32-3.47; *P* < .001) and faster time to death (HR, 3.80; 95% CI, 3.72-3.89; *P* < .001) were also observed among individuals with a mental disorder compared with those without a mental disorder or physical disease; although the effect sizes were smaller than those for individuals with a physical disease, they were substantial. Among individuals who died, the average number of years lived per person was 1.1 years shorter among those with a mental disorder compared with those without a mental disorder or physical disease. These associations were observed in men, women, and all age groups (Figure 3; eTable 1 in the Supplement). We observed a stronger association between mental disorders and mortality in the most recently born cohort compared with the earliest born cohort (eg, men born in 1968-1978, RR, 3.49 [95% CI, 3.28-3.71]; HR, 3.49 [95% CI, 3.29-3.71]; men born in 1928-1937, RR, 2.01 [95% CI, 1.86-2.16]; HR, 2.56 [95% CI, 2.37-2.76]); however, the increase in effect size was not linear across age groups.

Physical diseases were overrepresented among individuals with a mental disorder. A total of 33 153 of 103 929 individuals (31.9%) with a mental disorder also experienced a physical disease during the observation period, which exceeded the population-wide prevalence of physical disease (470 169 of 2 349 897 individuals [20.0%]). Associations with mortality were strongest among individuals with both a mental disorder and a physical disease (RR, 5.12 [95% CI, 5.02-5.22]; *P* < .001; HR, 5.92 [95% CI, 5.81-6.04]; *P* < .001) (Figure 3; eTable 1 in the Supplement).

### Mental Disorders and Physical Diseases

**Figure 4.**
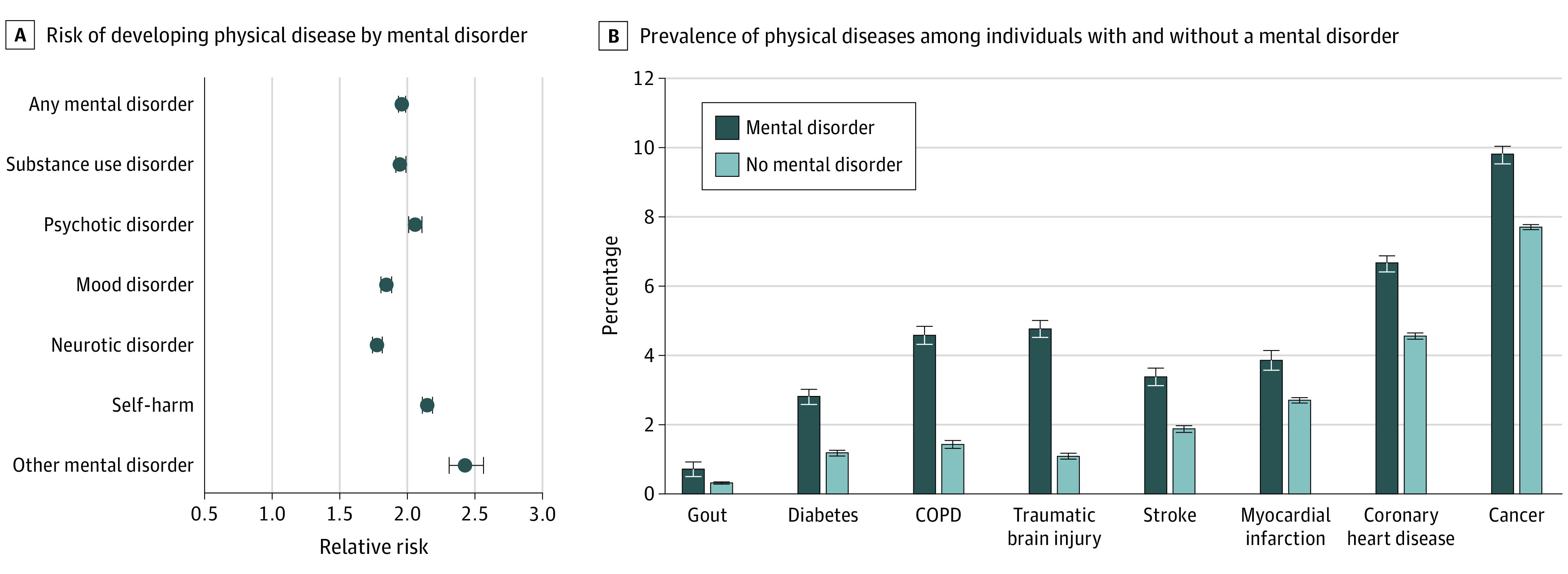
Specificity of Mental Disorder and Physical Disease Associations A, Mental disorders of many types were associated with subsequent physical diseases. Other mental disorder category includes physiological-disturbance, personality, developmental, behavioral, and unspecified disorders. Error bars indicate 95% CIs. B, Individuals diagnosed with a mental disorder were subsequently diagnosed with many different types of physical diseases across the observation period. Prevalence estimates for individuals with mental disorders across the 30-year period include all physical disease diagnoses received after the first mental disorder diagnosis; physical disease diagnoses that predated the first mental disorder diagnosis were excluded. Prevalence estimates for individuals without mental disorders across the 30-year period include all physical disease diagnoses received during the observation period. Risk ratios for estimates are available in eTable 4 in the Supplement. Error bars indicate SEs. COPD indicates chronic obstructive pulmonary disease.

After accounting for individuals who received a physical disease diagnosis before their index mental disorder diagnosis, those with a mental disorder remained at an increased risk of developing a subsequent physical disease (RR, 1.96; 95% CI, 1.93-1.99; *P* < .001), and they were more likely to develop physical diseases more quickly (HR, 2.33; 95% CI, 2.30-2.36; *P* < .001) (Table; Figure 4A; eTable 2 in the Supplement). Among individuals with a physical disease, those with a mental disorder developed the physical disease an average of 2.0 years earlier than those without a mental disorder (eResults in the Supplement). This association was observed for men, women, and individuals in all age groups (Table). Consistent with the pattern of associations for mortality, the associations between mental disorders and physical diseases were stronger among the youngest group compared with the oldest group. For example, among men born in 1968-1978, the RR of developing a physical disease was 2.48 (95% CI, 2.37-2.60) and the HR was 2.66 (95% CI, 2.55-2.78); among men born in 1928-1937, the RR of developing a physical disease was 1.25 (95% CI, 1.18-1.32) and the HR was 1.89 (95% CI, 1.81-1.97) (Table; eTable 2 in the Supplement).

**Table.** Associations Between Mental Disorders and Physical Diseases in the New Zealand Population^**a**^

Birth period	No. (% men)	Risk of developing physical disease			
		Men		Women	
		RR (95% CI)	HR (95% CI)	RR (95% CI)	HR (95% CI)
1968-1978	638 514 (51.0)	2.48 (2.37-2.60)	2.66 (2.55-2.78)	2.33 (2.21-2.45)	2.43 (2.31-2.56)
1958-1967	601 503 (50.8)	2.12 (2.03-2.20)	2.37 (2.28-2.46)	2.50 (2.40-2.61)	2.72 (2.61-2.84)
1948-1957	500 670 (50.8)	1.86 (1.79-1.93)	2.22 (2.14-2.30)	2.45 (2.35-2.56)	2.85 (2.74-2.97)
1938-1947	356 235 (50.5)	1.48 (1.42-1.55)	1.98 (1.91-2.06)	2.05 (1.96-2.15)	2.52 (2.42-2.62)
1928-1937	252 978 (50.0)	1.25 (1.18-1.32)	1.89 (1.81-1.97)	1.50 (1.42-1.58)	2.05 (1.97-2.14)

Abbreviations: HR, hazard ratio; RR, risk ratio.

^a^ Models controlled for physical diseases that were diagnosed before the index mental disorder. Counts were randomly rounded to a base of 3 per the confidentiality rules of Statistics New Zealand.

After controlling for history of physical disease, individuals diagnosed with substance use, psychotic, mood, neurotic, and all other mental disorders and individuals who engaged in self-harm remained more likely than those without a mental disorder to develop a subsequent physical disease. Associations were similar across psychiatric conditions (RRs, 1.78-2.43; *P* < .001 for all comparisons) (Figure 4A).

### Health Care Use and Costs

Relative to individuals without a mental disorder, those with a mental disorder were subsequently diagnosed with a greater number of different physical diseases, even after accounting for preexisting physical disease (IRR, 2.00; 95% CI, 1.98-2.03; *P* < .001) (Figure 4B). This multimorbidity was reflected in their interactions with the health care system; after accounting for preexisting physical diseases, individuals with a mental disorder accumulated a greater number of hospital admissions (IRR, 2.43; 95% CI, 2.39-2.48; *P* < .001), spent more days in the hospital for physical health problems (IRR, 2.70; 95% CI, 2.62-2.79; *P* < .001), and incurred higher governmental costs for the treatment of physical diseases (*b* = 0.115; 95% CI, 0.112-0.118; *P* < .001). The lifetime health care costs per person were 12.2% higher among individuals with a mental disorder relative to those without a mental disorder.

## Discussion

In this population register analysis of 2.3 million New Zealand citizens who were followed up across 3 decades, we found that individuals with mental disorders developed subsequent chronic physical diseases at younger ages and died at younger ages compared with individuals without mental disorders. These longitudinal associations were observed across different psychiatric disorders, across men and women, and across the life span, and they remained after accounting for preexisting physical diseases. Individuals with mental disorders were not only more likely to develop subsequent physical diseases, but they also had a greater number of different physical disease diagnoses and hospitalizations, longer hospital stays for physical disease treatment, and higher treatment costs.

Several implications can be noted. First, ameliorating mental disorders in early life might have implications for improving health, extending population life span, and mitigating health care costs. Associations with physical diseases were observed across individuals with different mental disorders, suggesting that ameliorating any mental disorder could be associated with health benefits in later life. Behavioral treatments that target common risk mechanisms across disorders (eg, cognitive behavioral and transdiagnostic approaches for emotion regulation^26^) might have the additional benefit of improving later physical health and longevity. However, the mechanisms that underlie the associations between different mental disorders and physical diseases might also differ. For instance, substance use disorders can cause liver damage and lung cancer, mood disorders may increase inflammation, and neurotic disorders might lead to high blood pressure. Future research is warranted to characterize the shared and distinct pathways of risk for physical diseases across mental disorders.

Second, mental disorders might be risk markers rather than risk factors for physical health problems. We empirically documented the temporal order in which mental disorders and physical diseases occur and addressed reverse causation via covariate control for preexisting physical diseases; however, we could not exclude all alternative explanations for these associations. The associations might reflect a general susceptibility to worse health. They may also be associated with the receipt of pharmaceutical treatments for mental disorders; however, most adverse pharmacologic effects (eg, metabolic disturbances) have been associated with the receipt of antipsychotic medications,^27,28^ and we observed associations across disorders that are not typically treated with antipsychotic medications, including substance use disorders, neurotic disorders, and self-harm behavior. Even if mental disorders do not directly cause physical diseases, they are salient early warning signs of later physical health problems, with implications for treatment delivery.^29^ Our findings support the integration of physical disease screening and prevention into the treatment of mental disorders. For instance, mental health professionals could provide psychoeducation to patients regarding their risks of developing later disease and could implement interventions designed to change health-associated behavior. Efforts have been made to incorporate mental health resources within medical settings (eg, primary care mental health integration programs within the US Veterans Health Administration^30^). Extending such efforts into the broader community could yield larger public health benefits.

Third, our results contribute to the increasing debate over the mortality gap among individuals with mental disorders. Some studies have suggested that the physical health of individuals with mental disorders has been worsening over time,^8,15,21,31^ while other studies have not consistently observed higher mortality in more recently born cohorts.^14,32^ We found that the associations of mental disorders with physical diseases and mortality were stronger among the most recently born cohort compared with the earliest born cohort. These findings are potentially consistent with a widening mortality gap and align with the results of a previous study, which indicated that the mortality gap between individuals with serious mental illness and the general population in New Zealand is comparable with the mortality gap found in other high-income countries.^33^ However, these associations might also reflect the possibility that younger individuals are more likely than older individuals to die of diseases and complications associated with mental health or that, in older cohorts, individuals who survive are selectively healthier than those in younger cohorts. Our analysis cannot resolve these questions, but it does reveal that worse mental health is associated with the risk of developing subsequent physical health problems among younger, not just older, populations.

Research following up the current findings (eg, studies of the factors underlying the association of mental disorders with subsequent physical diseases and longitudinal follow-up studies of physical health outcomes among participants in clinical trials of mental health treatments) presents opportunities for collaborative discussions among researchers and clinicians across scientific fields. Mental disorders, age-associated physical diseases, and mortality have largely been examined separately within the fields of psychiatry, geriatric medicine, and demography. Interdisciplinary research can inform prevention and treatment.

### Limitations

This study has several limitations. First, results are specific to 1 nation and 1 health care system. However, associations between mental disorders and early mortality have been observed across a range of countries, including the US,^15^ and the association between mental disorders and physical diseases was documented in Denmark across a 15-year follow-up period.^9^ We expanded on the Denmark study by using a 30-year follow-up period, accounting for preexisting physical diseases, and incorporating information about costs to society and government. Second, we accessed public rather than private hospital records. However, only an estimated 5% of New Zealand hospitalizations occur in private hospitals, and most of those hospitalizations are for elective surgeries.^34^

Third, inpatient hospital records will not capture most of the mental disorders that are less severe and are treated on an outpatient basis (or that do not prompt treatment seeking). For instance, in the Dunedin Study,^35^ a representative birth cohort of New Zealand citizens, for every individual with an administrative record of a psychiatric hospitalization by age 38 years, 20 individuals have a record of a psychiatric medication prescription. Underdiagnosis was likely less of a problem for physical diseases, as the dehospitalization movement has centered primarily on mental health treatment.^36^ In addition, the association between mental disorders and physical diseases has been identified using outpatient treatment records as well as inpatient hospital records.^9^

Fourth, we focused our analysis on chronic age-associated physical diseases, as these illnesses are most likely to be associated with disability and mortality. It remains to be determined whether the associations observed in the present study extend to unintended harm, injuries, and acute conditions. Fifth, results may vary based on historical differences in diagnostic practices. However, we observed associations between mental disorders and physical diseases among individuals who were born up to 50 years apart.

## Conclusions

There is an increasing need for improvements in mental health services and psychological treatment research.^37^ Prevention and intervention researchers responding to this need have a potential opportunity to improve not only the mental health of younger individuals but also the physical health of older individuals.^38^ Ameliorating mental disorders in early life could be associated with benefits for population health and longevity and reductions in the social and governmental costs associated with physical diseases as citizens age.

## Notes

Supplement. **eMethods 1.** Ascertainment of Mental Disorders and Physical Diseases **eMethods 2.** Measuring Physical Health Problems, Health Care Use, and Accumulated Costs **eMethods 3.** Random Matching Procedure **eResults.** Time to Physical Disease Among Individuals With and Without a Mental Disorder **eTable 1.** Associations Between Mental Disorders, Physical Diseases, and Mortality by Age and Sex **eTable 2.** Associations Between Mental Disorders and Subsequent Physical Diseases Derived Using the 5/25 Approach **eTable 3.** Hazard Ratios for Associations Between Mental Disorders, Physical Diseases, and Mortality Across Varying Time Intervals **eTable 4.** Risk Ratios for Differences in the Prevalence of Physical Diseases Among Individuals With and Without a Mental Disorder **eReferences**
