# Supplementary material for: Longitudinal Associations of Mental Disorders With Physical Diseases and Mortality Among 2.3 Million New Zealand Citizens
Source: JAMA Netw Open. 2021 Jan 13;4(1):e2033448. doi: 10.1001/jamanetworkopen.2020.33448 (PMC7807295; doi:10.1001/jamanetworkopen.2020.33448)
Supplement: Supplement. — eMethods 1. Ascertainment of Mental Disorders and Physical Diseases eMethods 2. Measuring Physical Health Problems, Health Care Use, and Accumulated Costs eMethods 3. Random Matching Procedure eResults. Time to Physical Disease Among Individuals With and Without a Mental Disorder eTable 1. Associations Between Mental Disorders, Physical Diseases, and Mortality by Age and Sex eTable 2. Associations Between Mental Disorders and Subsequent Physical Diseases Derived Using the 5/25 Approach eTable 3. Hazard Ratios for Associations Between Mental Disorders, Physical Diseases, and Mortality Across Varying Time Intervals eTable 4. Risk Ratios for Differences in the Prevalence of Physical Diseases Among Individuals With and Without a Mental Disorder eReferences [file jamanetwopen-e2033448-s001.pdf]

# Supplementary Online Content

Richmond-Rakerd LS, D'Souza S, Milne BJ, Caspi A, Moffitt TE. Longitudinal associations of mental disorders with physical diseases and mortality among 2.3 million New Zealand citizens. *JAMA Netw Open*. 2021;4(1):e2033448. doi:10.1001/jamanetworkopen.2020.33448

**eMethods 1.** Ascertainment of Mental Disorders and Physical Diseases

**eMethods 2.** Measuring Physical Health Problems, Health Care Use, and Accumulated Costs

**eMethods 3.** Random Matching Procedure

**eResults.** Time to Physical Disease Among Individuals With and Without a Mental Disorder

**eTable 1.** Associations Between Mental Disorders, Physical Diseases, and Mortality by Age and Sex

**eTable 2.** Associations Between Mental Disorders and Subsequent Physical Diseases Derived Using the 5/25 Approach

**eTable 3.** Hazard Ratios for Associations Between Mental Disorders, Physical Diseases, and Mortality Across Varying Time Intervals

**eTable 4.** Risk Ratios for Differences in the Prevalence of Physical Diseases Among Individuals With and Without a Mental Disorder

**eReferences**

This supplementary material has been provided by the authors to give readers additional information about their work.

© 2021 Richmond-Rakerd LS et al. *JAMA Network Open*.

## **eMethods 1.** Ascertainment of Mental Disorders and Physical Diseases

Mental disorders and physical diseases were ascertained using records of diagnoses made in public hospitals, based on the International Classification of Diseases, 9th Revision (ICD-9, for admissions between July 1988 – June 1999) and 10th Revision (ICD-10, for admissions between July 1999 – June 2018).

## Mental disorders

We obtained information about nine broad categories of mental disorders using the primary diagnoses for each hospital admission, based on the ICD-10 codes listed below. In order to achieve consistency across diagnostic schemes, corresponding diagnoses in the ICD-9 were ascertained using mapped codes provided by the New Zealand Ministry of Health. Back-mapping code is hosted at the following website: https://moffittcaspi.trinity.duke.edu/research-topics/statistical-code

For analysis purposes, physiological-disturbance, personality, developmental, behavioral, and unspecified disorders were grouped in one category.

| **Mental disorders** | **Abbreviated names** | **ICD-10** |
|---|---|---|
| Mental and behavioral disorders due to psychoactive substance use | Substance use disorders | F10 – F19 |
| Schizophrenia and related disorders | Psychotic disorders | F20 – F29 |
| Mood disorders | Mood disorders | F30 – F39 |
| Neurotic, stress-related, and somatoform disorders | Neurotic disorders | F40 – F48 |
| Syndromes associated with physiological disturbances and physical factors | Physiological-disturbance disorders | F50 – F59 |
| Disorders of adult personality and behavior | Personality disorders | F60 – F69 |
| Pervasive and specific developmental disorders | Developmental disorders | F80 – F89 |
| Behavioral and emotional disorders with onset usually occurring in childhood and adolescence | Behavioral disorders | F90 – F98 |
| Unspecified mental disorders | Unspecified mental disorders | F99 |

We also obtained information about self-harm using external cause codes for events of intentional self-harm (ICD-9: E950 – E959, ICD-10: X60 – X84). We excluded events of undetermined intent. Self-harm was always recorded, regardless of whether the hospital admission included a primary diagnosis of another mental disorder. If the admission included a primary diagnosis of another mental disorder, individuals were categorized as meeting criteria for both self-harm and the accompanying mental disorder.

## Physical diseases

We obtained information about eight chronic and age-related physical diseases, which comprise the diseases included in the Chronic Conditions Data Dictionary maintained by the New Zealand Ministry of Health (available at http://archive.stats.govt.nz/browse_for_stats/snapshots-of-nz/integrated-data-infrastructure/idi-data/chron-condn-sig-health-evt-data.aspx#gsc.tab=0). The dictionary documents procedures for ascertaining chronic conditions from New Zealand's health registers. We coded each disease using the primary diagnoses for each hospital admission (and procedure codes for coronary heart disease), based on the ICD-9 and ICD-10 codes provided in the data dictionary. The codes are listed below. Where ICD-9 codes were not provided, we used mapped codes provided by the New Zealand Ministry of Health. Back-mapping code is hosted at the following website: https://moffittcaspi.trinity.duke.edu/research-topics/statistical-code.

Per the procedures outlined in the data dictionary, both coronary heart disease and myocardial infarction were ascertained using ICD-10 code I21 and ICD-9 code 410. If these codes were given as the primary diagnosis, individuals were diagnosed with both conditions. Because coronary heart disease was also ascertained via procedure codes, occasionally a different physical disease was coded as the primary diagnosis for the same hospital admission. In these cases, individuals were diagnosed with both conditions.

© 2021 Richmond-Rakerd LS et al. *JAMA Network Open*.

| Physical diseases | ICD-10 | ICD-9 |
|---|---|---|
| Coronary heart disease[a] | Primary diagnoses:<br>I20 – I25, Z95.1, Z95.5<br><br>Procedure codes:<br>3530400, 3530500, 3531000, 3531001, 3531002, 3849700, 3849701, 3849702, 3849703, 3849704, 3849705, 3849706, 3849707, 3850000, 3850001, 3850002, 3850003, 3850004, 3850300, 3850301, 3850302, 3850303, 3850304, 3863700, 9020100, 9020101, 9020102, 9020103 | Primary diagnoses:<br>410 – 414, V45.81, V45.82<br><br>Procedure codes:<br>36.01, 36.02, 36.03, 36.04, 36.05, 36.06, 36.07, 36.10, 36.11, 36.12, 36.13, 36.14, 36.15, 36.16 |
| Gout | M10 | 274 |
| Chronic obstructive pulmonary disease | J40, J41.0, J41.1, J41.8, J42, J43.0, J43.1, J43.2, J43.8, J43.9, J44.0, J44.1, J44.8, J44.9 | 490, 491.0, 491.1, 491.20, 491.21, 491.8, 491.9, 492.0, 492.8, 496 |
| Diabetes | E10, E11, E13, E14, O24.0, O24.1, O24.2, O24.3 | Mapped codes |
| Cancer | C00 – C96, D45 – D47 | Mapped codes |
| Traumatic brain injury | S06 | 800 – 801.9, 803 – 804.9, 850 – 854 |
| Stroke | I60 – I64 | 430 – 432, 433.01, 433.11, 433.21, 433.31, 433.81, 433.91, 434.01, 434.11, 434.91, 436 |
| Myocardial infarction | I21 | 410 |

[a]Following the procedures given in the New Zealand Ministry of Health's Chronic Conditions Data Dictionary, procedure codes (in addition to primary diagnoses) were used to ascertain coronary heart disease.

© 2021 Richmond-Rakerd LS et al. *JAMA Network Open*.

**eMethods 2.** Measuring Physical Health Problems, Health Care Use, and Accumulated Costs

In addition to ascertaining specific physical-disease diagnoses, we measured four indicators of physical-health problems and associated healthcare involvement. (For all measures, we considered only the eight physical diseases included in the current study.)

1. **Number of different conditions.** We counted the number of different physical diseases that individuals accumulated during the observation period.

2. **Number of hospital admissions.** We counted the number of hospital admissions for physical diseases that individuals accumulated during the observation period.

3. **Total length-of-stay.** Total length-of-stay was defined as the number of calendar days from admission to discharge (same-day discharges = 0 days) across all admissions for physical diseases during the observation period.

4. **Total healthcare cost.** Total healthcare cost was estimated by summing "costweights" for each admission for a physical disease during the observation period. The raw measure was skewed and was therefore log-transformed for analyses.[a]

   Costweights are estimated based on the length-of-stay and complexity of the admission, where a costweight of 1 indicates an "average-cost" admission, a costweight of 0.5 costs half as much, etc. To assign costs equivalently across the observation period, costweights were multiplied by the unit purchase cost for the final fiscal year (2017/18: $4,921.16NZD).[1] Costweights were not available prior to 1999.

We aimed to ascertain the lifetime accumulation of physical-health problems, healthcare use, and costs following a mental disorder. Therefore, for individuals with a mental disorder, we included all physical-disease admissions that occurred after admission for their index mental disorder. Physical diseases that preceded the mental disorder were excluded from calculations.

[a]We report the association between mental disorder and total healthcare cost as an unstandardized regression coefficient (see **Results** in main text). Because the outcome is log-transformed, the unstandardized coefficient can be exponentiated to derive an estimate of percent change.

© 2021 Richmond-Rakerd LS et al. *JAMA Network Open*.

**eMethods 3.** Random Matching Procedure

We assessed whether mental-disorder hospitalizations between July 1988-June 2018 were associated with subsequent physical-disease hospitalizations during the period. Our analysis needed to account for the different duration of observation time among cases (those with a mental disorder, who were observed from their first mental-health hospitalization, which could occur at any time during the study period) and controls (those without a mental disorder, whose observation time was the full 30 years). Failing to account for differing observation periods could lead to biased estimates, because controls would have a greater opportunity to develop a physical-disease diagnosis than cases.

To address this problem, we randomly assigned observation periods to controls to match the observation periods of cases, based on the distributions of admission dates for cases' mental-disorder hospitalizations. For example, suppose .53%, .37%, and .41% of cases had their first mental-disorder hospitalization in July 1992, March 1998, and October 2015, respectively. In this scenario, we would randomly assign .53% of controls to have their observation period start in July 1992, .37% to have their observation period start in March 1998, and .41% to have their observation period start in October 2015. Importantly, we only recorded physical-disease hospitalizations during these truncated observation periods, ignoring all hospitalizations prior to the randomly-assigned starting months. We took this approach for each of the 360 months from July 1988 to June 2018, and so created a cohort of controls with the same distribution of observation periods as cases (**Figure 1** in main text).

Finally, to account for potentially different distributions of mental-disorder hospitalizations across age, we used birth cohort-specific distributions of start dates among cases to randomly assign start dates to controls. That is, the distribution of start dates among cases born in 1928-37 was used to randomly assign start dates to controls born in 1928-37, the distribution of start dates among cases born in 1938-47 was used to randomly assign start dates to controls born in 1938-47, etc.

© 2021 Richmond-Rakerd LS et al. *JAMA Network Open*.

**eResults.** Time to Physical Disease Among Individuals With and Without a Mental Disorder

We compared the mean time-to-physical disease (time-to-event) among individuals with versus without a mental disorder. Among individuals who developed a physical disease during the observation period, those with a mental disorder developed the disease 2.02 years earlier, on average, than those without a mental disorder (mean time-to-event: mental disorder = 9.02 years, no mental disorder = 11.04 years). The difference in mean time-to-physical disease between groups was similar after adjusting for pre-existing physical disease, sex, and birth year (difference = 1.67 years; adjusted mean time-to-event: mental disorder = 6.76 years, no mental disorder = 8.43 years).

© 2021 Richmond-Rakerd LS et al. *JAMA Network Open*.

**eTable 1.** Associations Between Mental Disorders, Physical Diseases, and Mortality by Age and Sex

## Relative risks

| Age band | Men | Women |
|---|---|---|
| | **Only mental disorder** | |
| Born 1968-78 | 3.49 [3.28, 3.71] | 4.41 [4.03, 4.83] |
| Born 1958-67 | 4.16 [3.92, 4.41] | 5.15 [4.74, 5.59] |
| Born 1948-57 | 3.96 [3.72, 4.22] | 5.29 [4.87, 5.73] |
| Born 1938-47 | 3.09 [2.88, 3.32] | 4.15 [3.83, 4.50] |
| Born 1928-37 | 2.01 [1.86, 2.16] | 2.60 [2.42, 2.78] |
| | **Only physical disease** | |
| Born 1968-78 | 4.00 [3.81, 4.19] | 14.03 [13.19, 14.92] |
| Born 1958-67 | 5.52 [5.33, 5.71] | 16.54 [15.82, 17.29] |
| Born 1948-57 | 4.84 [4.72, 4.97] | 13.16 [12.72, 13.62] |
| Born 1938-47 | 3.34 [3.27, 3.41] | 7.19 [7.00, 7.38] |
| Born 1928-37 | 2.02 [1.98, 2.06] | 2.93 [2.87, 2.98] |
| | **Both mental disorder and physical disease** | |
| Born 1968-78 | 5.25 [4.77, 5.77] | 11.90 [10.51, 13.48] |
| Born 1958-67 | 7.11 [6.63, 7.64] | 15.40 [14.15, 16.76] |
| Born 1948-57 | 6.50 [6.14, 6.88] | 14.14 [13.23, 15.13] |
| Born 1938-47 | 4.31 [4.09, 4.53] | 7.73 [7.31, 8.18] |
| Born 1928-37 | 2.23 [2.14, 2.34] | 3.16 [3.02, 3.31] |

## Hazard ratios

| Age band | Men | Women |
|---|---|---|
| | **Only mental disorder** | |
| Born 1968-78 | 3.49 [3.29, 3.71] | 4.34 [3.97, 4.75] |
| Born 1958-67 | 4.27 [4.03, 4.53] | 5.21 [4.80, 5.65] |
| Born 1948-57 | 4.19 [3.94, 4.47] | 5.56 [5.12, 6.03] |
| Born 1938-47 | 3.48 [3.24, 3.73] | 4.58 [4.22, 4.96] |
| Born 1928-37 | 2.56 [2.37, 2.76] | 3.35 [3.13, 3.59] |
| | **Only physical disease** | |
| Born 1968-78 | 4.01 [3.82, 4.20] | 14.16 [13.32, 15.06] |
| Born 1958-67 | 5.62 [5.43, 5.82] | 17.72 [16.95, 18.53] |
| Born 1948-57 | 5.04 [4.91, 5.18] | 15.09 [14.58, 15.61] |
| Born 1938-47 | 3.62 [3.55, 3.70] | 8.94 [8.71, 9.18] |
| Born 1928-37 | 2.38 [2.34, 2.42] | 4.04 [3.96, 4.11] |
| | **Both mental disorder and physical disease** | |
| Born 1968-78 | 5.25 [4.78, 5.77] | 11.84 [10.46, 13.41] |
| Born 1958-67 | 7.36 [6.85, 7.90] | 16.24 [14.92, 17.68] |
| Born 1948-57 | 6.92 [6.53, 7.32] | 16.05 [15.01, 17.17] |
| Born 1938-47 | 4.93 [4.69, 5.19] | 9.51 [8.98, 10.06] |
| Born 1928-37 | 2.75 [2.63, 2.88] | 4.36 [4.16, 4.57] |

Notes. The reference group for all comparisons is individuals without a mental disorder or a physical disease during the observation period. Models controlled for the total number of hospitalizations that individuals accumulated during the observation period. 95% confidence limits are in brackets. The larger associations among women than men are partly attributable to the higher mortality rate among men with no diagnosis (**Figure 3A,B** in main text), which might partly reflect that men are less likely than women to seek treatment, or basic gender differences in mortality.

© 2021 Richmond-Rakerd LS et al. *JAMA Network Open*.

## **eTable 2.** Associations Between Mental Disorders and Subsequent Physical Diseases Derived Using the 5/25 Approach

We used random-matching and weighting to account for differences in observation time (opportunity to develop a physical disease) among individuals with and without a mental disorder (method described in **eMethods 3** and **Figure 1** in main text). To test the robustness of this approach, we re-estimated the relative risks for associations between mental disorders and physical diseases by considering only mental disorders diagnosed during the first five years of the observation period, and physical diseases diagnosed in the subsequent 25 years ("5/25 approach"). As shown below, the estimates were very similar to those obtained using the matching approach.

| | | **Men** | | **Women** | |
|---|---|---|---|---|---|
| **Age bands** | **N (% men)** | **Matching** | **5/25** | **Matching** | **5/25** |
| Born 1968-78 | 638,514 (51.0) | 2.48 [2.37, 2.60] | 2.14 [1.97, 2.32] | 2.33 [2.21, 2.45] | 2.23 [2.04, 2.44] |
| Born 1958-67 | 601,503 (50.8) | 2.12 [2.03, 2.20] | 2.04 [1.91, 2.17] | 2.50 [2.40, 2.61] | 2.52 [2.35, 2.70] |
| Born 1948-57 | 500,670 (50.8) | 1.86 [1.79, 1.93] | 1.84 [1.74, 1.96] | 2.45 [2.35, 2.56] | 2.48 [2.33, 2.65] |
| Born 1938-47 | 356,235 (50.5) | 1.48 [1.42, 1.55] | 1.49 [1.40, 1.59] | 2.05 [1.96, 2.15] | 1.99 [1.86, 2.12] |
| Born 1928-37 | 252,978 (50.0) | 1.25 [1.18, 1.32] | 1.16 [1.07, 1.26] | 1.50 [1.42, 1.58] | 1.37 [1.27, 1.49] |

Notes. Estimates are relative risks [and 95% confidence intervals]. Models controlled for physical diseases diagnosed before the index mental disorder. Ns were randomly rounded to a base of three, per the confidentiality rules of Statistics New Zealand.

© 2021 Richmond-Rakerd LS et al. *JAMA Network Open*.

**eTable 3.** Hazard Ratios for Associations Between Mental Disorders, Physical Diseases, and Mortality Across Varying Time Intervals

To evaluate potential time differences in the associations between mental disorders, physical diseases, and mortality across the observation period, we calculated hazard ratios for the associations at five-year intervals, in the total study population. As shown below, although mental disorders and physical diseases were associated with a faster time-to-death at all intervals, the relative magnitude of the hazard ratios across groups varied at different lengths of follow-up. This can also be seen in **Figure 3C-E**.

| | **Time interval (length of observation)** | | | | | |
|---|---|---|---|---|---|---|
| | **5 years** | **10 years** | **15 years** | **20 years** | **25 years** | **30 years** |
| **Only mental disorder** | 4.54 [4.19, 4.92] | 4.45 [4.24, 4.67] | 4.07 [3.92, 4.22] | 3.72 [3.61, 3.83] | 3.60 [3.51, 3.69] | 3.80 [3.72, 3.89] |
| **Only physical disease** | 3.89 [3.76, 4.03] | 3.43 [3.35, 3.50] | 3.28 [3.23, 3.34] | 3.34 [3.30, 3.39] | 3.76 [3.72, 3.80] | 4.69 [4.65, 4.73] |
| **Both mental disorder and physical disease** | 4.08 [3.54, 4.72] | 4.08 [3.79, 4.38] | 3.92 [3.74, 4.11] | 3.85 [3.72, 3.98] | 4.31 [4.20, 4.42] | 5.92 [5.81, 6.04] |

Notes. Estimates are hazard ratios [and 95% confidence intervals]. Models controlled for sex, birth year, and the total number of hospitalizations that individuals accumulated during the observation period.

© 2021 Richmond-Rakerd LS et al. *JAMA Network Open*.

**eTable 4.** Risk Ratios for Differences in the Prevalence of Physical Diseases Among Individuals With and Without a Mental Disorder

| **Physical disease** | **Prevalence (%) among individuals with a mental disorder** | **Prevalence (%) among individuals without a mental disorder** | **Risk ratio** |
|---|---|---|---|
| Gout | 0.72 | 0.33 | 2.18 |
| Diabetes | 2.82 | 1.17 | 2.41 |
| COPD | 4.58 | 1.40 | 3.27 |
| Traumatic brain injury | 4.79 | 1.09 | 4.39 |
| Stroke | 3.39 | 1.88 | 1.80 |
| Myocardial infarction | 3.86 | 2.70 | 1.43 |
| Coronary heart disease | 6.64 | 4.57 | 1.45 |
| Cancer | 9.77 | 7.71 | 1.27 |

Notes. Prevalence estimates are also shown in **Figure 4B** in the main text.

© 2021 Richmond-Rakerd LS et al. *JAMA Network Open*.

## eReferences

1. New Zealand Ministry of Health. Weighted Inlier Equivalent Separations. https://www.health.govt.nz/nz-health-statistics/data-references/weighted-inlier-equivalent-separations

© 2021 Richmond-Rakerd LS et al. *JAMA Network Open*.
